# The Goldberg–Huxley model of the pathway to psychiatric care: 21st-century systematic review

**DOI:** 10.1192/bjo.2023.505

**Published:** 2023-06-23

**Authors:** Peter Huxley, Anne Krayer, Rob Poole, Alicja Gromadzka, Daniel Lai Jie, Sadia Nafees

**Affiliations:** School of Medical and Health Sciences, Bangor University, Bangor, UK; School of Psychology, Bangor University, Bangor, UK

**Keywords:** Pathway to care, common mental disorder, community prevalence, Goldberg–Huxley model, consultation rates

## Abstract

**Background:**

The classification of mental disorders used to be based only on people seen by hospital psychiatrists. In fact, most people with a mental disorder were, and are, not seen by psychiatrists because of decisions made prior to psychiatric consultation. The first description of this ‘pathway’ to care and its levels and filters was published by Goldberg and Huxley in 1980.

**Aims:**

To conduct a review of papers relevant to the application of the Goldberg–Huxley model in the 21st century.

**Method:**

Systematic review (PROSPERO registration CRD42021270603) of the pathway to psychiatric care in the 21st century. The review concentrates on community surveys and passage through the first filter (consultation in primary care or its equivalent). Ten databases were searched for papers meeting the defined inclusion criteria published between 2000 and 2019 and completed on 15 February 2020.

**Results:**

In total, 1824 papers were retrieved, 137 screened fully and 31 included in this review. The results are presented in a table comparing them with previous research. Despite major social, economic and health service changes since 1980, community prevalence and consultation rates remain remarkably consistent and in line with World Health Organization findings. Passage through the first filter is largely unchanged and there is evidence that the same factors operate internationally, especially gender and social parameters.

**Conclusions:**

The Goldberg–Huxley model remains applicable internationally, but this may change owing to an increasingly mixed mental health economy and reduced access to primary care services.

Until the late 1970s classification of mental disorders was based only on people seen by hospital psychiatrists. In fact, most people with a mental disorder were, and are, not seen by psychiatrists because of decisions made prior to psychiatric consultation, by the patient and their family doctor and others outside the hospital setting. The first description of this ‘pathway’ to care and its levels and filters was published by Goldberg and Huxley in 1980. Early in the 21st century, Singh & Grange (2006) argued that ‘in the era of clinical governance and quality assurance, understanding pathways to care is a crucial first step in ensuring improved clinical decision-making and effective service delivery’ (p. 81).^1^ In this spirit, we decided to update the literature on the Goldberg–Huxley (1980) model of the pathway to psychiatric care^2^ for the period 2000 to 2020, using a systematic review. This paper reports the results.

The model of the pathway to psychiatric care for people with common mental disorders (largely depression and anxiety) first published in 1980 is shown in Fig. 1 of that volume.^2^

This model described five levels, with a filter between each of the levels. Level 1 is morbidity due to common mental disorders within the community. The filter between level 1 and level 2 (primary medical care) involves decisions about seeking a medical consultation. The second filter is the primary care physician's detection of a disorder. This stands between level 2 and level 3 ‘conspicuous psychiatric morbidity’, which imply different levels of medical concern. The third filter is referral from primary care to level 4, which is specialist mental health services. Within specialist secondary care there is a further, fourth, filter, which is the decision to admit to in-patient care. This is level 5. In the latter part of the 20th century, a number of researchers contributed to the development of the model and reported on prevalence and consultation rates for common mental disorders at the different levels of the model.^3–5^

In the original work^2^ the median estimates for the 12-month prevalence of common mental disorders were 250 (per 1000 at risk per year) at level 1 (i.e. of random samples of everyone in the community) and 230 at level 2 (i.e. of those consulting their general practitioner, GP), of whom 140 (of the 230) were recognised as having a disorder. Only 17 of those with a recognised disorder reached a mental health professional, an observation repeated in a second book.^6^ The original estimates of contact with GPs were based on the ‘optimistic’ assumption that the prevalence at level 2 was only slightly lower than that at level 1. When simultaneous measures were made at both levels^6^ (p. 34) it became clear that patients with more severe symptom scores (or several diagnoses) were up to twice as likely to consult as those with lower scores, a finding replicated many times around the world since.^7–9^ A number of factors were identified that increased the chance of passing through the first filter. These include being female, being widowed, divorced or separated, and unemployment or other threatening life events. As well as providing an update on current prevalence and consultation figures we will also update the factors that contribute to an easier passage through the first filter, namely the decision to seek help for a common mental disorder.

In the first book we were able to find only five surveys that had used standardised research interviews in community settings.^2^ In the second book we added six further studies.^6^ The prevalence rate of disorder per 1000 population at risk was 164/1000 (males 121, females 202) when the 11 studies were taken together. We did report, but made no further comment on, the fact that the use of different psychometric instruments appeared to produce different figures. Lower rates were found when using the Present State Examination (PSE)^10^ than the Schedule for Affective Disorders and Schizophrenia and the Research Diagnostic Criteria (SADS-RDC)^11^ or the Clinical Interview Schedule (CIS).^12^ The major methodological improvements by the time of a third book about the model^13^ were the advent of large-scale representative population samples and the increase in the use of brief research diagnostic instruments.

Since the original work, a number of studies have made useful observations on the model. For instance, Tansella & Williams^14^ pointed out the heterogeneity of the data sources in the original model and argued, rightly, for contiguous data sources, that is from the same place and at the same time. To a considerable extent this advice has been followed in subsequent research.^6,15^

In 2006, Issakidis & Andrews^8^ applied the model to Australian data. They pointed out that a number of studies reported rates of morbidity at the higher levels of the model (i.e. levels 3–5), which is not the subject of the present paper. Only 22% of the Australian population met criteria for a mental disorder. Clinical factors were strongly associated with use of different care sectors, with physical health indicators important determinants of access to primary care in general, and mental health indicators important determinants of access to mental health care in both the primary and specialist sectors. Compared with younger age groups, people over 55 years of age were more likely to access the general primary care sector but were less likely to report mental health consultations in the primary care setting. Those living in rural areas were less likely to report access to the primary care sector than those living in metropolitan areas. Unlike most other reports up to that time, the authors looked at the use of private psychiatrists. Private psychiatrists and allied health professionals were consulted by 1.6% and 2.7% of the population respectively. They point out that although those accessing private and public out-patient care were clinically similar groups, those accessing private care were more likely to be employed and to have an independent income than those reporting public sector care only. People living in rural areas had lower consultation rates with private psychiatrists than those living in urban areas. These findings tended to support the idea that, where there is a functional state-funded mental health service in a high-income country, the activity of the private sector can be ignored as it is small. However, the findings are now quite old and there has been in a significant change in private provision in some countries, especially the UK. After many years of austerity economics, mental health services struggle to meet demand and the private sector has grown. It can no longer be assumed that private sector activity is insignificant. Furthermore, private provision is largely available in conurbations. In rural areas, it is hard to access private mental healthcare. Private care is not evenly distributed and if it is not considered, this may lead to spurious findings.

Usefully, Issakidis & Andrews also comment on some limitations of the model: ‘It does not focus explicitly on complex passages through the health-care system, passages back through the model, or the by-passing of various levels and filters, and several filters can be interpreted in a number of ways’. They make the important point, which remains relevant, that self-reporting of consultations is methodologically unreliable and may result in an underestimate of actual consultations. Later research included the upper levels and filters.^16–20^ These are not considered further in this review beyond pointing out that such research suggests that the original findings seriously underestimated the provision needed at level five.^21–23^

The model's estimate of prevalence at level 1 has been widely cited as showing that ‘one in four people have mental illness’. There has been a great deal of confusion and misreporting of this finding. Much of this is due to a failure to distinguish between different types of prevalence (such as point, period and lifetime), the failure to understand the difference between symptoms and disorders and a failure to compare like with like.^24^ As we will discuss later, taken together with aspects of the Andersen model^25^ (where a diagnosis of disorder is taken to indicate a need for treatment), this has contributed to the unmet need debate and to the concept of a ‘treatment gap’.^26^

It must be recognised that anxiety symptoms are ubiquitous and mostly short lived. The symptom severity threshold for mental disorder has always been controversial and, more recently, the construct of discrete mental disorders has been contested.^27^ Concerns over medicalisation of distress are not confined to the fringes: the former Chair of the DSM-IV Task Force has been highly influential in the debate.^28^ This illustrates that all diagnostic systems depend on a degree of judgement in assessing severity, which is unsatisfactory. The trouble with ‘needing treatment’ as a criterion is that ‘needing’ is not self-evident or easily defined.

In the present study, we have conducted a systematic search for papers containing findings about the use of the model, or those that appear to report rates of common mental disorders (anxiety and depression) at levels 1 and 2. Owing to the enormous number of papers published over the 20 years to 2020, we excluded papers reporting condition-specific rates, such as agoraphobia or post-traumatic stress disorder. If we had included them, the review would have been unworkably large and comparison with the findings in the first two books would not have been possible, as those original studies did not include rates for specific conditions. Studies that examine passage through the filters reveal the extent to which different clinical and sociodemographic characteristics facilitate or restrict movement to the next level. Later in this paper we summarise these findings.

## Method

### Search details

The review was registered with PROSPERO in 2021 (CRD42021270603).

We began with an internet search for the Goldberg–Huxley model from the first publication in 1980. We then searched electronic databases for published studies that used the model where data collection took place between January 2000 and December 2019 and for epidemiological studies that appeared to examine pathways to care. The end of 2019 was chosen as the cut-off point to exclude studies with findings that were affected by the COVID-19 pandemic. Studies conducted after that date suggest an increase in the community prevalence rate of common mental disorders,^29^ although these findings are contested.^30^

The following electronic databases were searched: MEDLINE via EBSCO*host*, PsycInfo via ProQuest, APA PsycNet, Embase and Science Direct via Elsevier, CINAHL via EBSCO*host*, Web of Science and Web of Knowledge, PubMed, Google and Google Scholar. No restrictions were placed on language of publication. The search focused on identifying original studies published in peer-reviewed journals, together with a search of grey literature. After piloting of search terms in consultation with Bangor University librarians, the following search strategy was used: psychiatric epidemiology OR mental health services AND (mental illness in the community OR pathway* to care OR common mental disorder) (NOT under 16 and above 65, dementia, randomised controlled trials, hospital or out-patient samples). Titles and abstracts were searched.

Identified papers were independently screened by two reviewers. They were retained where the abstract suggested that the paper might include data relevant to the first part of the model (that is, incidence and prevalence of common mental disorder, and consultation rates in primary care or its equivalent). At the next stage, inclusion decisions were again reviewed by two reviewers and studies were excluded if they reported data collected before the start of 2000, or had samples outside the 18–65 age range, or had a response rate of less than 60% or a sample size of less than 500.

**Fig. 1:**
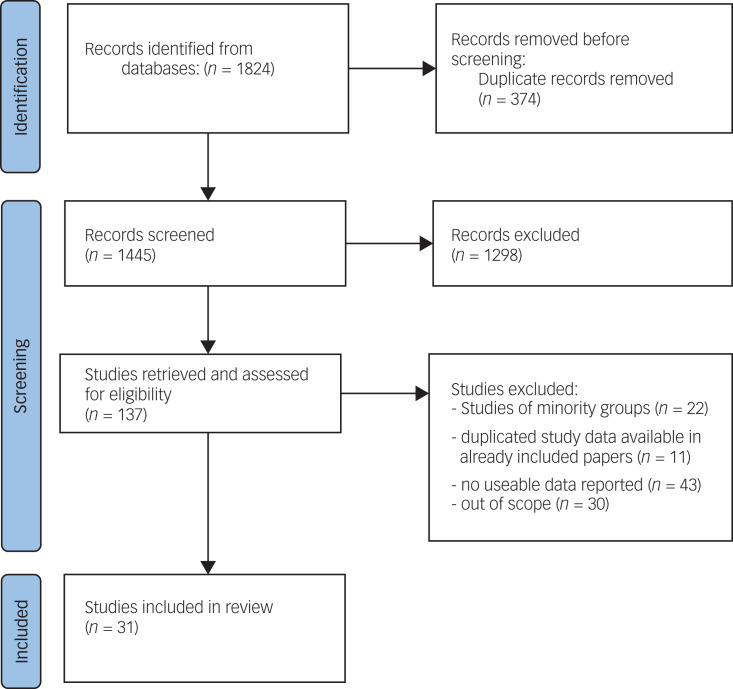
PRISMA flowchart for the identification and selection of studies.

Full-text versions of the remaining papers were scrutinised for extractable data. Duplicate papers from the same study were both retained if they reported on different variables of interest, but the sample ‘*n*’ was adjusted to avoid duplication in the overall sample size total given in this paper. The reasons for exclusion of papers are given in the PRISMA chart (Fig. 1). Within the papers included at this stage we identified those that used standardised measures of disorder at the community and primary care (or its equivalent) levels and papers in which the community-level data came from population-representative samples. Papers that reported findings from groups only, such as immigrants or ethnic minorities, either used data from wider representative surveys or conducted specific surveys of the groups concerned. We shall report on such studies in a separate paper.

Although the STROBE approach^31^ has been developed to assess the quality of the type of papers in this review, it has been pointed out that this does not result in a scale to assess quality. Even scales that have been produced from the STROBE guidance need additional elements to assess bias. Borges Migliavaca et al^32^ recommend considering the Joanna Briggs Institute (JBI) critical appraisal tool^33^ as the most suitable quality assessment for prevalence studies. Our quality ratings using the JBI tool were made by A.K. and P.H., with disagreement being resolved by a third author (R.P.).

Prevalence data from included papers were analysed using SPSS version 27 for Windows descriptive statistics, analysis of variance and independent-samples mean comparisons.

Finally, we re-examined papers with no usable prevalence data to see whether they reported variables related significantly to the permeability of the first filter (the initial decision to seek help in primary care).

## Results

No grey literature was identified. In total, 137 papers were subject to full review. Of these, some were excluded because they reported regression analysis without providing the figures for rates at levels 1 and 2. Some other papers reported no usable prevalence or consultation data. Taken together, reporting issues led to the exclusion of 43 of the 137 studies (31.4%). We re-examined these 43 to look for data relevant to the permeability of the first filter.

Duplication of data led to the exclusion of 11 of the remaining 94 papers (11.7%) and a further 30 (31.9%) were excluded as being out of scope (mainly owing to data collection prior to 2000). As described above, studies specifically examining minority groups were excluded, but will be analysed and reported in a separate paper. There were 22 such papers (23.4%). Thirty-one papers (32.9% of 94 and 22.6% of 137) were included the main analysis, although these were reporting on 34 samples.^18,34–61^ Some papers had titles that suggested they were reporting on an excluded group (e.g. elderly people) or a specific disorder (e.g. anxiety) when in fact they also reported data on the whole age or diagnostic range and so these were retained.

Using the JBI quality assessment for prevalence studies^33^ and scoring 1 for each satisfactory item resulted in normally distributed scores (mean 6.87, s.d. = 1.54; interquartile range IQR = 6–8). Only four studies (12.9%) received a maximum positive score; a further eight had only one negative rating (25.8%) (Supplementary material, available at https://dx.doi.org/10.1192/bjo.2023.505). The quality rating was unrelated to the prevalence results.

### Samples

Some included papers reported summaries of samples from different countries.^34,35^ A total of 34 separate samples were reported in the 31 papers. Our unduplicated aggregated sample size (excluding Chiu et al^44^) is 312 069. The mean individual sample size is 10 761 (s.d. = 13 894.3) and the median is 5201 (IQR = 2698.5–10 261.0).

### Measures

The most commonly used standardised assessment measure was the World Health Organization (WHO) Composite International Diagnostic Interview (CIDI)^62^ (*n* = 21 studies; 61.8%). The Clinical Interview Schedule – Revised (CIS-R)^12^ and the Alcohol Use Disorder and Associated Disabilities Interview Schedule-IV (AUDADIS-IV)^63^ were used in two studies each (*n* = 4; 11.8%) and nine other measures were used only once (26.1%). Twelve-month prevalence and consultation rates did not differ by instrument used.

### Lifetime service use

Lifetime service contact was usually determined through self-report, which is recognised to be unreliable.^23,64–66^ Only eight of the included papers reported on lifetime service use. Given the methodological problems, we report frequencies but offer no further analysis. Mean lifetime service use was 27.8% (s.d. = 15.13), with a range from 10.6% to 55.9%.

### Twelve-month prevalence and consultation rates

Table 1 shows that our prevalence figures are broadly comparable with WHO World Mental Health (WMH) study results (WHO results are excluded from our data-set in this comparison). The 2022 data from the WHO^67^ report a prevalence range from 10.9% in Africa to 15.6% in the Americas. The high-income country (HIC) rate is reported as 15.1% and the lower-middle-income (LMIC) rate as 11.6%. Our HIC rate is 14.5% and LMIC is 9.8%. The primary care consultation rate in the WHO WMH study ranged from a low of 2% in Nigeria to a high of 18% in the USA. In our sample, the lowest rate was in China (2.7%) and the highest in Europe (13.0%), a non-significant difference.

**Table 1:** Prevalence and consultation rates (per 1000 at risk per year): data from this study and World Health Organization (WHO) World Health Statistics^67^

	Community prevalence (level 1)	Population service use (level 2)	Service use – those with disorder (level 2+)
Samples in this study, *n*	15	16	14
Mean (s.d.)	13.8 (6.6)	7.9 (5.5)	20.7 (12.2)
Median	15.2	4.9	22.6
IQR – this study	7.5–18.9	4.1–14.1	6.3–33.7
IQR – WHO	9.1–16.9		
Range – this study	4.8–24.2	1.9–18.0	5.3–36.9
Range – WHO^a^	4.3–26.4	0.8–15.3	14.6–64.5

IQR, interquartile range.

a. Excluded both the highest and lowest four cases.

A number of papers examined changes in 12-month prevalence and consultation rates over time: in Canada between 2006 and 2014;^44^ in Japan between 2002 and 2015;^56^ and in Australia between 1997 and 2007.^58^ Chiu et al^44^ used administrative-linked data from a population of 11 million in Ontario to show that, although out-patient psychiatric consultation rates declined significantly over the 8 years, consultations with GPs remained stable. Ishikawa and colleagues^47^ showed that the prevalence rate for common mental disorders in Japan remained constant over the 13-year period. The consultation and treatment rates of people with a disorder increased over time, although neither increase was significant. Parslow and colleagues^54^ found that, although the proportion of people accessing any mental healthcare service within the previous 12 months increased significantly, from 12.4% to 21.4% over 10 years, the proportion accessing GP care for mental health problems did not increase. These three studies showed an essentially similar pattern of stability for both prevalence and consultation rates at the primary care level.

### Sensitivity analyses

We examined regional differences in prevalence rates. One-third of the samples were from Europe and just over one-third from the Americas. The remainder came from Africa and the Pacific Rim. South America and Africa show lower prevalence rates than other regions, but overall differences are not significant (*F* = 1.03, d.f. = 5, *P* = 0.42). There was no difference in our sample between LMICs and HICs in either prevalence rates (*t* == 0.54, d.f. = 14, *P* = 0.59) or level 1 consultation rates (*t* = 0.92, d.f. = 13, *P* = 0.37).

### Factors associated with the first filter

Several papers did not report prevalence figures but did undertake statistical analyses in relation to the permeability of the first filter, most often regression analysis, but also latent class analysis.^68^ These methods provide fairly robust findings because they control for the influence of other variables. This section is based on these papers dating from 2000 to 2019. As indicated earlier, the analysis here excludes studies that specifically investigated issues of ethnicity, immigration and refugee status.

One of the most consistently reported findings is that women are more likely to consult doctors when distressed.^69,70^ The rate is often double that of men (as we reported in 1980, p. 24).^2^ It is noteworthy that in our 2000–2019 studies this is a consistent cross-cultural finding, which is reported in Brazil,^37^ Canada,^35^ Germany^18^ and other parts of Europe,^71^ Japan,^47^ Tehran,^72^ in different ethnic groups in the USA^73–76^ and in Shanghai.^77^ Men are more likely than women to consult for substance misuse or impulse control disorders.^70,78^

Those with more severe illness or more than one diagnosis pass more easily through the first filter. This too is true in different cultures, including Australia,^79^ Italy,^36^ Brazil,^37^ the USA^74,76^ and Iran.^45^

A further common finding is that variables related to marital status make passage easier. Being divorced, widowed or single is associated with easier passage to the second level. This is reported in Australia,^8,35^ Brazil,^37^ Japan,^47^ in parts of Europe^71^ and in Shanghai.^80^

Papers reporting age as a factor are few, but their findings are consistent. Middle-aged people have higher consultation rates than the young or the old. This has been reported in Australia,^8^ Korea^40^ and the USA.^78,80^

A small number of other factors have been reported to make passage through filters more likely. These include insufficient family support,^80,81^ lower income^38,47^ and higher education.^38,56,71^ Unemployment is also a factor and has been reported in several countries, including China,^77^ France,^81^ Korea,^40^ The Netherlands^38^ and the USA.^75^

There are studies showing that within services that are not funded by the state, the first filter is less permeable. This may occur where health insurance does not cover mental illnesses^82^ or where provision is dominated by private provision, as in Brazil.^37^ Permeability is enhanced where public assistance payments are readily available, as in Korea.^40^ There is some ambiguity over whether resources at levels 2–5 have an impact on provision and accessibility, but this debate is beyond the scope of the present paper.

Rural areas appear to have similar prevalence rates to urban areas, but consultation rates are lower. Lower rural consultation rates have been reported in The Netherlands^38^ and other parts of Europe,^71^ but not in South Africa.^60^ In Australia, rural area residents with common mental health problems are more likely to see a psychiatrist than a GP. This is thought to be related to service availability.^8^

Finally, since the model was first proposed in 1980, there has been an exponential growth in the availability of non-medical sources of help for people in distress in the community. This includes internet-based helping services and (telephone) help-lines. Some papers report internet use in their surveys, but commonly fail to report the results separately.^76,83^ Similarly, some studies collect complementary and alternative medicine (CAM) use in their surveys but again fail to report the frequency of use separately.^76^ Reports of CAM use for help with mental distress are inconsistent and vary by location. In Australia, Olesen and colleagues^84^ found that half of all adults who met the criteria for an affective or anxiety disorder in the previous 12 months reported use of non-practitioner-led support services. Six per cent of their sample used support services, including internet and face-to-face support groups and telephone counselling. As is found in traditional health services, those with a 12-month disorder used these services more frequently (approximately five-fold, a similar ratio to our results in Table 1). Burns & Tomita^74^ reported that 48.1% of individuals seeking formal healthcare for mental disorders in Africa had previously consulted traditional and religious healers. Earlier reports^85^ suggested that this choice is associated with delay in accessing formal mental health services. The average rate of use of CAM services by those in the community with symptoms is not dissimilar in Japan, the USA and Columbia, at between 20% and 28%. Although many people used these services for mental health reasons, they also used them for physical health reasons such as low back pain.^52^

## Discussion

Although there is heterogeneity in the results of the studies in this review (as observed in other reviews^73^), there is also a remarkable degree of consistency and stability in some aspects of the model, which was first proposed over 40 years ago. This is particularly remarkable because the total sample size in 1980 was relatively small, as there were few large-scale representative community samples.^2^ Furthermore, there was very limited use of standard diagnostic measures at the time.^2^

The prevalence and consultation rates found in the present review are within a similar range to the WHO studies (Table 1), which is perhaps to be expected given the large sample sizes involved. Also worthy of comment is the similarity between the original factors related to passage through the first filter and those reported here. Furthermore, even though international health and social systems have changed considerably, a similar set of important social and demographic factors are seen across time and very different countries. Moreover, family physician/general practice/primary healthcare are still the services most commonly used by distressed people^34^

The main methodological limitation of the evidence presented in this review is the reliance on self-report of mental illness. As others have indicated, under-reporting is likely.^8,9,45,68,71,86^ There is also inconsistency in the diagnoses that are included in the studies. In particular, some papers include substance misuse and impulse control disorders whereas others do not. In some cases, findings for separate disorders are presented, but in other studies they are conflated: respondents were asked about ANY mental health AND/OR substance misuse consultations. Reporting this information is important for comparative and cumulative research.

Self-reported mental health service use is not necessarily reliable in recall of details, such as frequency of use or the type of service provider/health professional. This is exacerbated by the lack of consistent definitions or typologies of mental health service provision. In some instances, ‘mental health services’ are defined broadly and conflate levels of the model.^40,87^ An agreed and generally applicable typology of mental health services would help in aggregating and comparing and would facilitate consistent reporting of the use of digital services^88–90^ and CAM. The evidence on the extent of the use of CAM is unclear, in part because of inconsistent reporting, but also owing to local cultural differences. These differences and reported increases require further exploration.^91,92^

Authors who report only regression analysis must, of necessity, have had access to prevalence or consultation rates in order to report on factors leading to passage through the first filter. It would be a great service if journal editors insisted that these data are reported in the paper or in supplementary material.

### Limitations

The exclusion criteria resulted in a reduced number of papers. It could be argued that data collected before the cut-off date of January 2020 but published later ought not to have been excluded. Similarly, the cut-off point of the start of the pandemic means another reduction in the available data. The main justification for both decisions is to reduce heterogeneity in what is widely accepted as a very heterogeneous research field. In the case of the end cut-off, we have already indicated that the effects of the pandemic on prevalence rates is contested and therefore we feel this is an additional justification for our approach. The other major excluded category is of studies that use regression analysis but do not report the associated prevalence figures. Although we excluded these from the prevalence analyses, we did include them with reference to passage through the first filter. This form of multivariate analysis controls for the influence of multiple variables of interest and therefore gives greater confidence in the variables identified as significant.

### Implications

Since the original model was proposed, there have been substantial changes in the organisation and delivery of services. These continue at an increasing pace around the world. Clarity about what constitutes mental health ‘services’ is not only important to understanding pathways to care, as Singh & Grange^1^ argue. It is also important to documenting and evaluating ‘help-shifting’ as service modalities change from more traditional provision, such as out-patient clinics, to newer forms or perhaps to less appropriate ones. In the UK, for instance, there is a new phenomenon of waiting lists for emergency care. Many in-patient services and crisis teams run such waiting lists. Pressures on the National Health Service (NHS), especially in primary care, have led to an unplanned system whereby hospital emergency departments are the *de facto* main point of access to mental health services, the effect of which may be the application of a single binary filter (‘does the patient need to be admitted?’). In Wales, Part 3 of the Mental Health (Wales) Measure 2010 created a right for Welsh residents to refer themselves to specialist mental health services, bypassing the Goldberg–Huxley filters. The increasingly mixed health economy and poor access to primary care have significantly undermined the primary care gatekeeping role, and in any case primary care services are not universal. New non-medical primary care provision must be taken into account, for example IAPT (Improved Access to Psychological Treatment teams in England) and consultation models where a consultant psychiatrist discusses patients with GPs without actually meeting the patients. The international impact of changes of this type is, as yet, largely absent from the literature. The implications of such changes on the future applicability and robustness of the model have yet to be explored. Another potential and contentious influence on prevalence rates are the varied steps taken to cope with the COVID-19 pandemic. The contested nature of this impact^29^^,^^30^ warrants much further research.

## Data Availability

Data availability is not applicable to this article as no new data were created or analysed in this study.

## Supplementary material

For supplementary material accompanying this paper visit http://doi.org/10.1192/bjo.2023.505. click here to view supplementary material
